# Mechanisms of NLRP3 priming in inflammaging and age related diseases

**DOI:** 10.1016/j.cytogfr.2020.08.003

**Published:** 2020-10

**Authors:** Anna Gritsenko, Jack P. Green, David Brough, Gloria Lopez-Castejon

**Affiliations:** a Lydia Becker Institute of Immunology and Inflammation, Division of Infection, Immunity and Respiratory Medicine, Faculty of Biology, Medicine and Health, University of Manchester, Manchester Academic Health Science Centre, Manchester, UK; b Lydia Becker Institute of Immunology and Inflammation, Division of Neuroscience and Experimental Psychology, Faculty of Biology, Medicine and Health, University of Manchester, Manchester Academic Health Science Centre, Manchester, UK

**Keywords:** NLRP3, Inflammasome, Priming, Inflammaging, Senescence, Aging

## Abstract

The NLRP3 inflammasome is a vital part of the innate immune response, whilst its aberrant activation drives the progression of a number of non-communicable diseases. Thus, NLRP3 inflammasome assembly must be tightly controlled at several checkpoints. The priming step of NLRP3 inflammasome activation is associated with increased NLRP3 gene expression, as well as post-translational modifications that control NLRP3 levels and licence the NLRP3 protein for inflammasome assembly. Increasing life expectancy in modern society is accompanied by a growing percentage of elderly individuals. The process of aging is associated with chronic inflammation that drives and/or worsens a range of age related non-communicable conditions. The NLRP3 inflammasome is known to contribute to pathological inflammation in many settings, but the mechanisms that prime NLRP3 for activation throughout aging and related co-morbidities have not been extensively reviewed. Here we dissect the biochemical changes that occur during aging and the pathogenesis of age related diseases and analyse the mechanisms by which they prime the NLRP3 inflammasome, thus exacerbating inflammation.

## Highlights

• The process of aging is associated with chronic inflammation termed inflammaging.
• Inflammaging drives a number of age related diseases.
• Biochemical changes driven by aging prime NLRP3 inflammasomes transcriptionally, post-transcriptionally and post-translationally.
• Enhanced NLRP3 inflammasome activation worsens neurodegenerative and metabolic conditions as well as cancer.

## Graphical abstract

**Figure:**
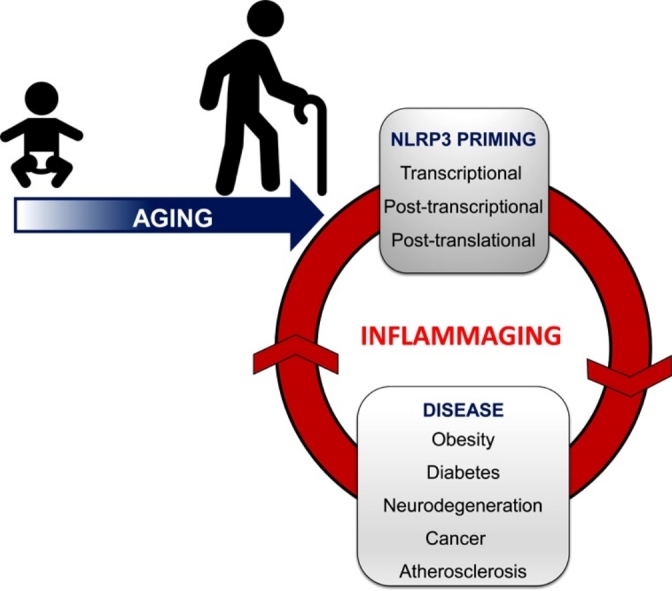

## 1 Introduction

**Fig. 1:**
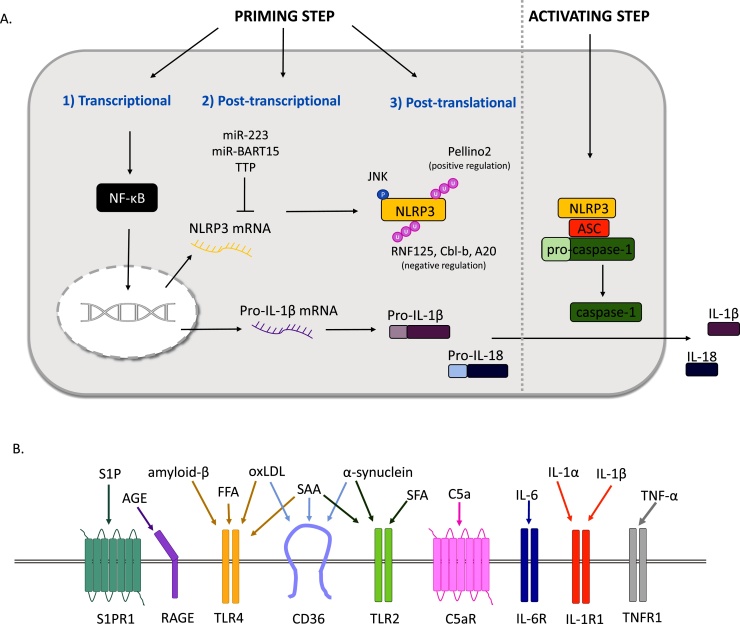
Recognition of sterile inflammatory factors by innate cell surface receptors primes NLRP3 for inflammasome activation. **A.** Sensing of priming signals regulate NLRP3 at different levels 1) transcriptional, 2) post-transcriptional and 3) post-translational prior to the second or activating step. Nuclear factor-κB (NF-κB); reactive oxygen species (ROS); sensor (NOD)-like receptor protein 3 (NLRP3); apoptosis-associated speck-like protein containing a CARD (ASC); Interleukin (IL); tristetraprolin (TTP). **B.** Sterile priming stimuli act upon a wide variety of cell surface receptors, with the same stimuli being able to target different receptors. Sphingosine-1-phosphate (S1P) receptor 1 (S1PR1); advanced glycation end product (AGE); receptor for AGEs (RAGE); free fatty acid (FFA); oxidised low density lipoprotein (oxLDL); toll like receptor 4 (TLR4); serum amyloid A (SAA); cluster of differentiation 36 (CD36); saturated fatty acid (SFA); toll like receptor 2 (TLR2); complement component 5a (C5a) receptor (C5aR); Interleukin-6 (IL-6) receptor (IL-6R); Interleukin-1 (IL-1) receptor 1 (IL-1R1); tumour necrosis factor-α (TNF-α) receptor 1 (TNFR1).

The innate immune system is our first line of defence against invading pathogens and is activated by pathogen and damage signals. To orchestrate an appropriate response, pattern-recognition receptors (PRRs) recognise pathogen-associated molecular patterns (PAMPs) or damage-associated molecular patterns (DAMPs) and mediate downstream inflammatory pathways [1]. Inflammasomes are multiprotein complexes in immune cells. The best studied inflammasome contains the sensor (NOD)-like receptor protein 3 (NLRP3), as well as an adaptor protein: apoptosis-associated speck-like protein containing a CARD (ASC) and the effector enzyme caspase-1. The current dogma in the field is that canonical NLRP3 inflammasome activation is a two-step process: priming (step 1) and activation (step 2) [2] (Fig. 1A). Priming is considered essential for expression of the NLRP3 gene, as well as the caspase-1 substrate pro-interleukin (IL)-1β that otherwise has low expression [3]. Priming also involves a range of post-translational modifications (PTMs) to NLRP3 that ‘licence’ the protein by allowing it to form the correct conformation for self-oligomerisation/ interaction with ASC to enable inflammasome assembly [4] or rescue it from degradation [5]. NLRP3 is able to respond to a range of activating stimuli including nigericin toxin, extracellular adenosine triphosphate (ATP), as well as lysosomal destabilisation agents such as silica and cholesterol crystals [6]. Upon inflammasome activation, oligomerised NLRP3 polymerises the adaptor protein ASC, recruiting pro-caspase-1, which undergoes proximity-dependent auto-activation and as a result cleaves pro-IL-1β and constitutively expressed pro-IL-18 into their mature forms. Concurrently, activated caspase-1 causes the cleavage of gasdermin-D (GSDMD) into N-terminal fragments that form lytic pores, thus targeting the cell for pyroptotic cell death and facilitating the release of mature IL-1β and IL-18 [7]. An alternative NLRP3 inflammasome has also been described in human monocytes, where prolonged exposure to priming signals can induce the release of IL-1β [8]. Through the binding to receptors on other cells, IL-1β and IL-18 initiate and propagate inflammatory responses to clear the threat. However, their aberrant release is responsible for the pathogenesis of a range of non-communicable diseases [9]. Indeed, the NLRP3 inflammasome is strongly linked to a range of age related ailments, including metabolic disorders and neurodegenerative diseases [10]. Mice deficient in NLRP3 have increased longevity and show reduced signs of aging e.g. thymic involution, inflammation and functional decline [11]. Here we review the different mechanisms of NLRP3 priming by gene upregulation and licencing by PTMs and focus on how these priming signals contribute to the pathogenesis of age related non-communicable diseases.

### 1.1 Transcriptional regulation of NLRP3 by priming signals

NLRP3 responds to a broad repertoire of stimuli, allowing it to combat a range of viral and bacterial infections such as adenovirus, influenza, *Staphylococcus aureus, Salmonella typhimurium, Listeria monocytogenes* and *Mycobacterium* [12]. These pathogenic stimuli are able to enhance NLRP3 inflammasome activation by upregulating its gene expression. In certain cells, *de novo* protein synthesis of NLRP3 in response to toll like receptor (TLR) agonists is required for NLRP3 activation. The engagement of TLR -2,-3,-4 and -7 by Pam3CysK4, Poly(I:C), LPS and R848 respectively has been described to upregulate NLRP3 expression through nuclear factor-κB (NF-κB) pathways, a step that is essential for subsequent NLRP3 activation in macrophages [3]. This overcomes the low protein levels of NLRP3 that are thought to limit its activation [13]. However, NLRP3 activation can also occur in the absence of infection. For example, IL-1α is an alarmin secreted by damaged cells, which through IL-1R1 can engage NF-κB dependent gene expression via MyD88 [14]. Similarly, tumour necrosis factor (TNF)-α acts through its receptor to prime NLRP3 for activation in macrophages, independently of MyD88 [15]. IL-1β is not constitutively expressed but contains a functional NF-κB binding site, allowing it to be strongly upregulated by NF-κB signalling. As IL-1β is an inducer of NF-κB signalling downstream of IL-1R1, a positive autoregulatory loop is created, allowing IL-1β to potentially prime cells in a paracrine manner [16] (Fig. 1B). Overall, the NF-κB binding site on the NLRP3 promoter is well characterised and converges many inputs from different priming factors to upregulate NLRP3 expression [3]. Other transcription factors such as Sp1, c-Myb, AP-1, and c-Ets [17], as well as sterol regulatory element-binding transcription factor 2 (SREBP2) [18] have been suggested to contribute to NLRP3 expression. Given the diversity of signals that prime NLRP3 inflammasome, it is not surprising that multiple transcription factors can bind and regulate expression of the NLRP3 gene, leading to the common outcome of increased transcription.

### 1.2 NLRP3 post-transcriptional regulation in response to priming signals

Tight regulation of NLRP3 is also apparent at the post-trancriptional level. The NLRP3 gene undergoes alternative splicing in the 5′-untranslated region (UTR) to generate three alternative splice forms, which have varying levels of promoter activity that exist within the human population [17]. The SNP (−1064 T) has been found in the NLRP3 gene of a mutation negative familial cold autoinflammatory syndrome (FCAS) patient. A construct with this SNP displayed significantly higher promoter activity [17], suggesting that increased NLRP3 expression is associated with an inflammatory disease phenotype that is independent of the well known NLRP3 gain of function mutations. It has been proposed that different cytokine milliue can differentially effect the expression of these isoforms, although the effect on inflammasome activation has not been explored. Alternative splicing further regulates the Leucine-rich repeat (LRR) domain of NLRP3 to generate a full length variant or one lacking exon 5 [19]. The isoform that lacks exon 5 loses an ability to bind NIMA-related kinase 7 (NEK7), which has recently been described as a factor for NLRP3 priming [20]. However, whether alternative splicing affects the ability of the cells to be primed has not been investigated.

Epigenetic modifications modulate gene activity without altering the DNA sequence [21] and are implicated in NLRP3 regulation through methylation and acetylation. For example, following *Mycobacterium tuberculosis* infection, the promoter region of the human NLRP3 gene is de-methylated, increasing NLRP3 expression and enhancing inflammasome activation [22].

It is becoming increasingly evident that innate immune mediators are also subjected to regulation by non-coding RNAs [21]. MicroRNAs (miRNAs) bind to the UTRs of transcripts to control mRNA stability and translation. miRNAs are often negative feedback mediators of the innate immune system by targetting a range signalling molecules downstream of TLR4 [23]. miR-223 has been described to suppress NLRP3 expression by binding to a conserved site in the 3′UTR of NLRP3, resulting in decreased inflammasome assembly [24]. miR-223 expression is constitutively high in NLRP3 inflammasome forming cells [24]. However, it has been revealed that miR-223 is upregulated in the blood and lung parenchyma of tuberculosis patients. It is therefore possible that priming signals mediate the levels of miRNAs that post-transcriptionally regulate NLRP3 (Fig. 1A). Viruses are known to exploit this machinery to inhibit the inflammasome, facilitating their own reproduction [25]. For instance, Epstein-Barr Virus (EBV) produces miR-BART15 that targets the same region as miR-223 to suppress inflammasome activation [26]. It has been recently revealed that the RNA binding protein Tristetraprolin (TTP) targets the AU-rich element of human NLRP3 3′UTR to repress NLRP3 expression, whilst knocking down TTP makes NLRP3 more sensitive to priming signals such as LPS and enhances the inflammasome response [27]. The same study reports alternative polyadenylation of NLRP3, producing a short 3′UTR isoform lacking TTP and miRNA-223 binding sites.

### 1.3 NLRP3 post-translational regulation in response to priming signals

As well as regulation by transcriptional and post-transcriptional mechanisms, NLRP3 is subjected to PTMs [4]. The addition/removal of various low weight molecular groups can modulate protein folding, stability, localisation and interaction with other proteins [28]. A number of PTMs have been proposed to regulate the NLRP3 inflammasome both at the priming and the activation step. Here we will focus on the NLRP3 control triggered by priming signals. Regulation of NLRP3 PTMs triggered by the activation step such as phosphorylation or ubiquitination have been previously extensively reviewed [29,30].

The post-translational regulation of NLRP3, mediated by priming signals, licences NLRP3 for subsequent activation by signal 2. For example, JNK1 mediates the phosphorylation of NLRP3 at Ser194 following LPS priming, promoting NLRP3 deubiquitination, self-association and inflammasome assembly [31]. The same can be said for changes in the ubiquitination status of NLRP3. Following a sub-lethal dose of LPS, E3 ligases RNF125 and Cbl-b sequentially polyubiqutinate NLRP3. RNF125 initiates K63 linked ubiquitination of the LRR domain, which is then bound by Cbl-b for K48 ubiquitination and degradation of NLRP3 by the proteasome. This prevents endotoxemia via NLRP3 and caspase-11 (non-canonical) dependent mechanisms [32]. The ubiquitin modifying enzyme A20 is a NF-κB inhibitor and also functions to restrict spontaneous NLRP3 inflammasome activation. In A20 deficient macrophages, NLRP3 assembles an active inflammasome in response to LPS alone in a RIPK3 dependent manner, thus losing its requirement for signal 2. In LPS treated macrophages, A20 was seen to form a complex with caspase-1, caspase-8, RIPK1, RIPK3, whilst in the absence of A20, these enzymes show increased association with pro-IL-1β [33]. On the other hand, E3 ubiquitin ligase Pellino2 promotes NLRP3 ubiquitination during the priming step and is required for inflammasome activation independently of mediating degradation [34] (Fig. 1A). Moreover, short LPS priming, through mechanisms independent of gene transcription, has been reported to deubiquitinate NLRP3 and licence it for inflammasome activation in bone marrow derived macrophages (BMDM)s [13]. These studies highlight that NLRP3 is regulated by PTMs as a consequence of priming signals, which is critical for inflammasome assembly.

Other PTMs have emerged as regulators of NLRP3 licencing, including acetylation. Acetylation of NLRP3 at K24 by the histone acetyltransferase KAT5 has been suggested to facilitate NLRP3 self-oligomerisation following treatment with inflammasome activators [35]. Moreover, inhibiting KAT5 activity blocks inflammasome derived IL-1β production *in vivo*. During aging, there is a decreased expression of the deacetylase sirtuin (SIRT)2. This enzyme has been shown to modify the NLRP3 protein by removing acetyl groups from K21 and K22, thus preventing NLRP3-NLRP3 and NLRP3-ASC interactions and inflammasome assembly [36]. Therefore, intrinsic changes in gene expression associated with aging lift the negative regulation of NLRP3 and licence it for inflammasome assembly.

## 2 Aging Associated Priming

**Fig. 2:**
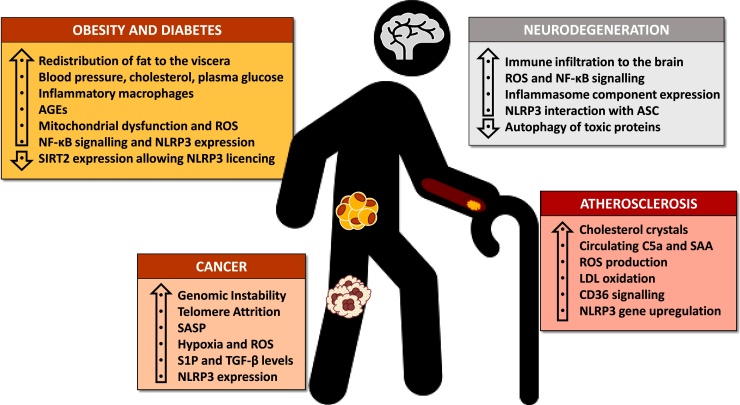
Biological changes that occur during inflammaging and the pathogenesis of age-related diseases and how these potentiate the NLRP3 inflammasome response. Nuclear factor-κB (NF-κB); reactive oxygen species (ROS); sensor (NOD)-like receptor protein 3 (NLRP3); apoptosis-associated speck-like protein containing a CARD (ASC); complement component 5a (C5a); serum amyloid A (SAA); low density lipoprotein (LDL); cluster of differentiation 36 (CD36); senescence associated secretory phenotype (SASP); sphingosine-1-phosphate (S1P); tumour growth factor-β (TGF-β); advanced glycation end product (AGE); sirtuin 2 (SIRT2).

The process of aging is associated with an increase in chronic, low grade inflammation that is referred to as inflammaging [37]. This manifests itself in enhanced severity of non-communicable conditions associated with aging e.g. neuroinflammatory disorders such as Alzheimer’s and Parkinson’s, cancer and cardiovascular diseases (CVD) such as atherosclerosis and diabetes (Fig. 2). The western diet and an increased incidence of obesity only add to the problem of chronic inflammation and multi-morbidity in the aging population [38].

Throughout the course of aging, the immune system becomes increasingly dysregulated. Some immune functions are decreased, for example macrophages from aged mice have a reduced ability to phagocytose necrotic cells, in comparison to those from young mice [39]. Moreover, human PBMCs from elderly donors display significantly lower autophagic markers [40], whilst overexpressing autophagosome formation protein Atg5 in mice extends lifespan compared to WT mice [41]. Decreased autophagy is believed to impair the ability to clear misfolded proteins and damaged organelles with age. In addition to impeded beneficial functions, aging is intimately associated with an inflamed state driven by cellular senescence [42], mitochondrial dysfunction and oxidative stress [43] and disease specific mediators such as saturated fatty acids, advanced glycation end products, oxidised low density lipoprotein (LDL), amyloid-β and α-synuclein that prime the NLRP3 inflammasome via various mechanisms (Fig. 1B). Aging also correlates with altered composition of the microbial community, resulting in intestinal ‘leakage’, inflammation and reduced macrophage function [44]. This was found to be mediated by high levels of TNF-α, a cytokine known to prime NLRP3. Moreover, aged mice have significantly higher levels of the bacterial cell wall component muramyl dipeptide (MDP) in the plasma [44], suggesting that PAMPs enter the circulation and may therefore prime for inflammasome activation. The levels of miRNAs that negatively regulate inflammatory pathways e.g. miR-223, which supress NLRP3 translation [24], increase with age [45] in an attempt to compensate for the accelerated inflammation during the progression of aging.

### 2.1 Senescence associated secretory phenotype

Aging is associated with, and may even be driven by, an accumulation of senescent cells [46]. Senescent cells are permanently arrested in the G1 or G2/M phase of the cell cycle. Stopping cell division after a certain number of cycles plays an important role in preventing malignant transformation [47]. Nevertheless, these cells remain metabolically active, and gain a secretory phenotype, termed the senescence‐associated secretory phenotype (SASP) that is overall pro-inflammatory. For example, persistent DNA damage or late passage induced senescent cells secrete large amounts of pro-inflammatory cytokines IL-6 and IL-8 [48]. Innate immunity plays a role in mediating senescence. The cGAS-STING pathway is important for recognising foreign DNA in innate immunity, but is also essential for DNA damage induced senescence and the expression of inflammatory mediators [49]. It has been reported that oxidative stress induced senescence, via cGAS-STING, activates NF-κB and drives NF-κB dependent genes such as IL-1β and IL-6 [50]. Indeed, NF-κB subunit p65 accumulates on the chromatin of senescent cells and is a master regulator of SASP related genes [51].

Various inflammatory intercellular signalling pathways initiated by SASP have been described. These include paracrine effects on neighbouring cells, orchestrated by IL-6 and IL-8 [52]. For example, studies have shown that IL-6 is sufficient to prime neutrophils for subsequent inflammasome activation with monosodium urate (MSU) [53]. A major cell type attracted by secretory factors from SASP cells are macrophages [54]. Although it has not been reported, it would make sense that pro-inflammatory cytokines from SASP cells recruit macrophages and prime the NLRP3 inflammasome. In murine lungs, aging significantly increases NLRP3, ASC, IL-1β and IL-18 expression [55]. The same study also used co-immunoprecipitation to reveal increased interaction between NLRP3 and ASC in lung homogenates of aged mice compared to young mice following reactive oxygen species (ROS) induced lung injury. IL-1β and IL-18 secretion is also increased in aged mice and is dependent on NLRP3. Moreover, NLRP3 deficiency protects mice from lung injury and fibrosis. In this context, macrophages from aged lungs showed elevated levels of mitochondrial ROS production, and treatment with mitoTEMPO, a mitochondrial targeted antioxidant, resulted in a significant decrease in IL-1β and IL-18 production in both young and aged macrophages [55]. As well as senescence associated macrophages, senescent fibroblasts themselves have been described to show increased expression of NLRP3 inflammasome components and also caspase-1 activation. Additionally, caspase-1 inhibition partially inhibited cell cycle arrest, whilst IL-1R1 inhibition did so fully, suggesting that IL-1β plays a role in reinforcing senescence and also highlights the role of IL-1α [56]. Taken together, this evidence suggests that aging associated SASP and oxidative stress provide mechanisms for both NLRP3 inflammasome priming and activation.

### 2.2 Obesity and associated diseases

The increasing incidence of obesity and complications associated with this disease account for a large proportion of the health burden in the western world [57]. Abdominal obesity, insulin resistance, hypertension, and hyperlipidemia are all characteristics of the metabolic syndrome, which is a major risk factor for the development of diabetes and cardiovascular complications such as atherosclerosis [58]. The incidence of metabolic syndrome is strongly correlated with age [59]. Indeed, aging is accompanied by a redistribution of body fat to the viscera [60], as well as an increase in systolic blood pressure (SBP), total cholesterol (TC), and fasting plasma glucose (FPG) [61] (Fig. 2).

In the pathogenesis of obesity, the growing body of adipose tissue undergoes metabolic, endocrine and immune changes. Excess nutrient availability is an initiator of mitochondrial dysfunction and ROS production [62]. In addition to this, aging too leads to an increase in mitochondrial DNA deletions [63], mitochondrial dysfunction and enhanced ROS generation [43]. Moreover, aging is associated with decreased expression of miRNA processing machinery in adipose tissue, for example Dicer. Knocking down Dicer in animal models causes increased sensitivity to oxidative stress and even reduces lifespan of the animals [64]. The effect of excess ROS on oxidative stress and subsequent inflammation is extensively described [43]. As well as facilitating step 2 of canonical NLRP3 inflammasome activation, ROS have emerged as a mediator of NLRP3 priming. In immortalised macrophages, NLRP3 expression requires priming by PAMPs such as LPS. In this setting, ROS inhibition dose-dependently inhibits the expression of NLRP3 and also prevents inflammasome activation when ROS is inhibited prior to LPS priming [65]. Obesity is also characterised by a chronic hypoxic state. Hypoxia, through NADPH oxidase function, leads to elevated ROS production [66] and can directly engage NF-κB gene expression and prime NLRP3 for inflammasome activation [67]. Macrophages subjected to hypoxia increase NLRP3 expression and prevent IL-1β from autophagic degradation, making hypoxic macrophages secrete higher levels of IL-1β [68]. Thus, both obesity and aging converge at the level of enhanced ROS production and associated inflammation, resulting in NLRP3 upregulation as well as inflammasome activation in a sterile setting.

#### 2.2.1 Diabetes mellitus

Obesity has strong implications in pancreatic β-cell dysfunction and insulin resistance that manifest as diabetes mellitus (DM) [69]. The increasing incidence of diabetes in the aging population is correlated with early onset of disability and mortability [70]. The biochemical changes that occur as a consequence of excess nutritition, insulin resistance and aging have been shown to mediate NLRP3 priming via various mechanisms (Fig. 2).

Aged mice exhibit enhanced TNF-α production that increases NLRP3 expression. As a result, aged mice show caspase-1 activation in myeloid cells within the adipose tissue, as well as enhanced serum IL-18 and impaired glucose tolerance [71]. Adipose associated macrophages increase in numbers in obese individuals and are the primary source of elevated pro-inflammatory factors such as TNF-α, iNOS and IL-6 [72]. The levels of free fatty acids (FFA) are elevated in obesity and have major signalling roles in obesity associated inflammation and insulin resistance [73]. FFAs activate NF-κB signalling, increasing TNF-α and IL-6 expression in both macrophages and adipocytes in a TLR4 dependent mechanism [74]. The same study reported that TLR4 deficiency protects mice from high fat diet (HFD) induced insulin resistance. Therefore, obesity associated FFAs create an inflammatory environment within adipose tissue and likely prime inflammasome components. Indeed, macrophages isolated from mice on a HFD showed increased IL-18, IL-1β and caspase-1 expression. Adipocytes co-cultured with these macrophages *in vitro* showed upregulated NLRP3 gene expression [75]. HFD rich in saturated fatty acid (SFA) also significantly upregulates NLRP3, caspase-1 and IL-1β expression in murine adipose tissue and primes the inflammasome for activation following ATP treatment [76]. Palmitic acid is a major dietary SFA that has been reported to cause the dimerization of TLR1 and TLR2, leading to increased pro-IL-1β expression, but also caspase-1 activation and IL-1β release from THP-1 cells and primary human monocytes [77].

As well as forming the canonical inflammasome, human monocytes can assemble an alternative inflammasome in response to prolonged TLR stimulation alone [8]. Apolipoprotein C3 (ApoC3) secretion is elevated in patients with type II diabetes [78] and its overexpression results in decreased adipose lipolysis and increased fatty acid uptake by adipose depots [79]. It was recently revealed that ApoC3 can activate the alternative NLRP3 inflammasome through the dimerization of TLR2 and TLR4 [80]. This results in IL-1β release but no GSDMD cleavage and pyroptosis, which are not features of the alternative inflammasome. Although no pyroptosis occurred in monocytes, ApoC3-mediated monocyte activation exacerbated kidney damage after unilateral ureter ligation. This study emphasises the importance of characterising the NLRP3 response in sterile diseases, where disease derived factors may prime and/or activate the canonical inflammasome or engage the alternative inflammasome.

Advanced glycation end products (AGEs) arise as a result of non-enzymatic glycation of plasma proteins due to hyperglycaemia [81]. Although AGEs accumulate during normal aging, their production is greatly increased in individuals suffering from obesity and diabetes [82]. Treatment of BV2 microglial cells with AGEs results in the activation of RAGE-ROCK-NF-κB pathway and the upregulation of NLRP3 expression [83]. Exposure to AGEs also leads to significant ROS production, which is a known activator of NLRP3 inflammasome, although this was not explored. A different study reports that mice administered with AGEs exhibit increased islet β-cell apoptosis and pancreatic damage, resulting in decreased glucose tolerance. These effects were ameliorated in NLRP3 KO mice, suggesting that NLRP3 inflammasome activation bridges the association between AGEs and pancreatic dysfunction. Moreover, pancreatic caspase-1 activation was blocked with ROS inhibitor, once again highlighting the role of ROS in inflammasome activation [84].

Serum amyloid A (SAA) is a liver derived acute phase protein which has been found to have immunological activity [85]. SAA levels positively correlate with aging [86] as well as the incidence of type II diabetes [87]. SAA causes a dose-dependent increase in IL-1β expression and primes the NLRP3 inflammasome for further activation with DAMPs in cultured mixed glia [88]. The priming functions of SAA are mediated by TLR2, TLR4 and CD36, as blocking these receptors lowers IL-1β gene expression [89]. The direct priming of NLRP3 by upregulating its transcription was shown in macrophages [90]. As well as priming, SAA is able to signal through the ATP receptor P2X_7_ [89] to induce intracellular ROS and cathepsin-B mediated NLRP3 inflammasome activation and IL-1β secretion [90].

It has recently been reported that NLRP3 is modified by acetylation and is deacetylated by SIRT2, which prevents NLRP3 inflammasome activation [36]. SIRT2 mediated deacetylation of NLRP3 inhibits aging and HFD associated inflammation and insulin resistance *in vivo*. However, the expression of SIRT2 decreases with age, allowing inflammasome mediated inflammation to persist [36]. This adds to the evidence that NLRP3 licencing by PTMs regulates its activation *in vivo* and suggests that the landscape of NLRP3 regulating enzymes changes with age, facilitating inflammasome activation.

#### 2.2.2 Atherosclerosis

Atherosclerosis is characterised by the accumulation of lipids and fibrous matter in the vasculature. Although atherosclerosis is largely a consequence of chronic vascular injury caused by elavated cholesterol, it has been suggested that age dependent loss of bone marrow derived vascular progenitor cells exacerbates this disease [91]. The profound inflammatory response including leukocyte recruitment and pro-inflammatory cytokine production [92] also plays a key role in disease progression. In particular, TNFR1 has been identified as a key mediator of aging dependent atherosclerosis in mice, whilst SNPs in human TNFR1 are significantly associated with CVD in elderly patients [93].

Although the NLRP3 inflammasome has been implicated in the pathology of atherosclerosis, disease manifestation in germ free mice is no different to normally housed mice, suggesting sterile priming and activation of the inflammasome [94]. Circulating cholesterol, specifically LDL, is correlated with disease severity and its oxidation (esterification or lipid peroxidation) initiates many of the inflammatory pathways [95]. CD36 is a scavenger receptor present in monocytes, macrophages, endothelial cells and adipocytes. Upon the recognition of oxLDL, CD36 functions to initiate foam cell formation and a number of intracellular cascades [96]. CD36 has been reported to prime the NLRP3 inflammasome *in vivo* [97]. Specifically, oxLDL upregulates IL-1β and NLRP3 gene expression, which does not occur in CD36, TLR4 or TLR6 KO macrophages. As well as priming, oxLDL can act as signal 2 for NLRP3 inflammasome activation. Apolipoprotein E (ApoE) KO mice fed a western diet have elevated IL-1β and IL-18 serum levels, as well as caspase-1 activation in plaques, which is significantly lowered in CD36, TLR4 and TLR6 KO. Therefore, CD36 complexed with TLR4 and TLR6 mediates both oxLDL induced priming and NLRP3 inflammasome activation [97]. Interestingly, whilst NLRP3 deficiency in ApoE KO mice does not reverse atherosclerosis progression [98], NLRP3 mediates systemic inflammation and pathogenesis in LDL receptor KO mice that are fed a western diet [99]. However, caspase-1/11 deficiency decreases the size of atherosclerotic lesions in mice as demonstrated by various studies [100]. Moreover, targeting IL-1β with canakinumab was shown to significantly lower the rate of recurrent cardiovascular events as part of the The Canakinumab Anti-inflammatory Thrombosis Outcome Study (CANTOS) trial [101].

Cholesterol crystals that appear in early atherosclerotic lesions can activate the NLRP3 inflammasome, causing caspase-1 cleavage and mIL-1β release in a process associated with lysosomal rupture [102]. The authors of this study went on to show neutrophil recruitment and the development of atherosclerotic lesions was dependent on IL-1, as well as NLRP3 and ASC *in vivo.* Another study reported that cholesterol crystals act as a priming signal by upregulating NLRP3, caspase-1 and IL-1β gene expression in primary human macrophages and even cause modest mIL-1β release in the absence of LPS [103]. Calcification of vasculature also plays a role in NLRP3 priming. Rat vascular smooth muscle cells (VSMC) that undergo calcium deposition following β-Glycerophosphate treatment have time dependent increase in expression of NLRP3, ASC and caspase-1 which was accompanied by caspase-1 cleavage and IL-1β release, suggesting that calcification can also activate the inflammasome [104].

Atherosclerosis is also mediated by the complement system. The complement component C5a is a highly inflammatory peptide that facilitates immune cell attraction, phagocyte activation and the release of oxidants and granule based enzymes [105]. Interestingly, the levels of circulating C5a become increased during normal aging [106]. In atherosclerosis, C5a induces acute complications such as plaque disruptions [107]. Cholesterol crystals employ C5a to upregulate pro-IL-1β and also cause complement dependent production of ROS that activates caspase-1, facilitating IL-1β maturation and release [108]. Whilst C5a enhances LPS induced expression of IL-1β in monocytes via p38, C5a supresses LPS mediated upregulation of IL-1β, NLRP3 and caspase-1 via PI3K in macrophages [109], highlighting differential requirements for priming between cell types. Overall, the evidence suggests that molecular patterns associated with atherosclerotic plaques can prime and activate the NLRP3 inflammasome, further driving inflammation and disease progression.

### 2.3 Neurodegenerative disorders

Aging is the most dominant risk factor for the majority of neurodegenerative diseases, which tend to develop irreversibly, resulting in a large socioeconomic burden and great personal cost to patients, often with no effective treatment available [110]. The central nervous system (CNS) was once considered an immunoprivileged site due to its separation from the periphery with the blood brain barrier (BBB), but it is now appreciated that the immune system mediates many of the brain’s homeostatic processes and can drive disease pathogenesis [111]. In fact, the BBB becomes increasingly ‘leaky’ with age, allowing non-specific transport of neurotoxic proteins to readily enter the brain and potentially contribute to neurodegeneration [112]. Microarray analysis of aging brains reveals significant upregulation of inflammatory genes and microglial activation markers compared to young individuals [113]. The development of Alzheimer’s Disease (AD), Parkinson’s Disease (PD) and Multiple Sclerosis (MS), through varying mechanisms, have been evidenced to prime and activate the NLRP3 inflammasome, which subsequently mediates further inflammation and damage. One feature common to these diseases is decreased autophagy, which has been reported to cause neurodegeneration in mice [114]. Reduced autophagy lowers the clearance of toxic protein aggregates in the brain [115], and also rescues kinases involved in NF-κB signalling from selective degradation [116], thus potentially allowing NLRP3 transcriptional priming (Fig. 2). Understanding the mechanisms of NLRP3 activation in these diseases is critical, as the NLRP3 inflammasome has been shown to be necessary for age related inflammation, astrogliosis and functional decline in a mouse model [11].

#### 2.3.1 Alzheimer’s disease

The most common form of dementia is AD, with aging being the main risk factor for its development and subsequent irreversible functional decline [117]. Many of the brain’s immune functions are carried out by microglia, which in their resting state survey the environment for infection or tissue damage with supressed NLRP3 inflammasome functions [118]. In AD, microglia gain a more pro-inflammatory phenotype [119]. Quantitative PCR analysis of hippocampal gene expression of IL-1β, NLRP3 and ASC reveals upregulation in AD patients compared to healthy controls [113]. Treatment of microglia with soluble amyloid-β precursor protein also upregulates pro-IL-1β expression [120]. Although the exact mechanisms of NLRP3 priming in AD are unclear, it has been reported that in a cell free system, amyloid-β oligomers and fibrils interact with NLRP3 and induce its interaction with ASC. Moreover, in transfected HEK cells, amyloid-β oligomers drive the release of IL-1β, suggesting that amyloid-β alone is sufficient to prime and activate the inflammasome [121]. TLR4 is thought to mediate this signalling as TLR4 or MyD88 deficient cell lines treated with amyloid-β oligomers show impaired IL-1β secretion [122]. NLRP3 inflammasome activation may be an important driver of disease progression. In a mouse model of AD, NLRP3 or caspase-1 deficiency facilitate microglia to gain an anti-inflammatory phenotype, enhance amyloid-β clearance, decrease amyloid-β deposition and protect mice from the loss of spatial memory [123]. Activated microglia co-cultured with primary neocortical neurones elicit neuronal tau phosphorylation and a reduction in synaptophysin: a marker of synaptic terminals. This is IL-1β dependent, as IL-1 receptor antagonist, as well as anti-IL-1β antibody, attenuate this effect [120]. Overall, there is strong evidence for amyloid-β driven inflammation and NLRP3 inflammasome priming and assembly, which has been implicated in the development of AD pathology.

#### 2.3.2 Parkinson’s disease

PD arises from the loss of neurones in substantia nigra, causing striatal dopamine deficiency, and also through the formation of misfolded aggregates of the pre-synaptic protein α-synuclein [124]. A growing number of studies suggest a link between the NLRP3 inflammasome and the pathogenesis of PD, and in particular how changes that occur in PD can prime and/or activate the NLRP3 inflammasome. It has been reported that α-synuclein treated microglia *in vitro* results in upregulated expression of TLR1,2,3 and 7. Moreover, the mRNA of signalling molecules MyD88 and NF-κB was also elavated, as well as that of IL-1β and TNF-α [125]. Although NLRP3 was not investigated, this data indicates microglial activation and a switch to a pro-inflammatory phenotype. This was built upon by a study that identified TLR2 as the receptor required for α-synuclein mediated IL-1β gene induction in human monocytes [126]. NLRP3 was also upregulated by α-synuclein treatment. As well priming the monocytes, α-synuclein caused NLRP3 inflammasome activation and IL-1β release through its phagocytosis and downstream mechanisms involving lysosomal distabilisation and ROS production. Further work revealed the role of CD36 on murine microglia in regulating α-synuclein mediated microglial activation, TNF-α production and ROS generation [127]. In PD patients, the gene expression of NLRP3, ASC and caspase-1 in PBMCs are indeed elevated. Plasma concentration of IL-1β is also significantly increased and positively correlate with both α-synuclein levels and the severity of disease [128]. Thus, α-synuclein can carry out both inflammasome priming and activating functions.

#### 2.3.3 Multiple sclerosis

The median age of patients with MS is 55–59 years [129]. Although MS is not considered a disease of the elderly, the increasingly aging population means that patients live with this disease for decades following onset in young adulthood. Moreover, it has been suggested that aging related inflammation plays a role in chronic diffuse demyelination of MS [130]. This is supported by the fact that aging negatively affects disease prognosis [131]. MS is an autoimmune disorder where CNS inflammation demyelinates neurones, which has been extensively linked to NLRP3 inflammasome activation in macrophages and dendritic cells [132]. In human MS samples, the expression of NLRP3, caspase-1 and IL-1β are significantly upregulated compared to healthy tissue [118]. Moreover, the expression of caspase-1 in PBMCs correlated with the number of new MS lesions in patients [133]. NLRP3 mRNA is also elevated in a mouse model of MS: autoimmune encephalomyelitis (EAE). Here, NLRP3 deficient mice exhibit reduced immune cell infiltration and pro-inflammatory cytokine production, resulting in significantly delayed course and severity of pathology [134]. Similar findings were reported in ASC and caspase-1 deficient mice [135,136]. Thus, it is plausible that the initiation of MS upregulates inflammasome components, which then function to drive the progression of the disease.

### 2.4 Cancer

The incidence of cancer is strongly correlated with age, with age related factors such as genomic instability, telomere attrition, dysregulated nutrient sensing, senescence and chronic inflammation increasing the risk of cancer [137] (Fig. 2). Inflammation is a key driver of both development and progression of carcinogenesis. Inflammatory factors that increase the risk of cancer include bacterial and viral infections, but also sterile factors such as obesity, alcohol, tobacco and autoimmune diseases [138]. During carcinogenesis, aberrant signalling due to oncogenic mutations drive tissue microenvironments to gain a more inflammatory phenotype. Sites of tumours are largely orchestrated by immune cells that play indispensable roles in proliferation, survival and migration [139].

Tumour associated macrophages (TAMs) are commonly found in tumour microenvironments, where they orchestrate inflammation and promote angiogenesis and metastatic spread [140]. Macrophages are the main producers of IL-6 and are therefore mediators of IL-6 driven inflammation and tumour promotion in colitis associated cancer [141]. Sphingosine-1-phosphate (S1P) is a bioactive lipid metabolite that has a range of physiological functions downstream of its receptor. Intracellularly, S1P can facilitate TNF receptor-associated factor 2 (TRAF2) mediated canonical NF-κB activation pathway [142]. S1PR1 deficiency on TAMs surrounding murine breast tumours prevents pulmonary metastasis and tumour lymphangiogenesis [143]. Interestingly, transcription analysis reveals decreased NLRP3 expression levels in S1PR1 deficient macrophages. In both mouse and human macrophages, S1PR1 inhibition results in reduced NLRP3 mRNA levels and IL-1β production. Increased NLRP3 expression by macrophages is correlated to reduced survival rates in patients with invasive breast cancer [143].

As the tumour outgrows its blood supply, the environment becomes increasingly hypoxic. Hypoxia is associated with significantly higher IL-1β secretion by TAMs due to increased stability of hypoxia inducible factor 1α (HIF‐1α) [144]. Through the stimulation of NF-κB genes, hypoxia has been shown to transcriptionally prime NLRP3 and pro-IL-1β in both macrophage and prostate cancer cell lines and potentiate inflammasome activity [67]. NLRP3 upregulation can be seen across a range of cancers. In a mouse model of pancreatic carcinoma, NLRP3, IL-18 and IL-1β are upregulated inside infiltrating macrophages. This was mirrored in human pancreatic ductal adenocarcinoma, where tumour infiltrating monocytes expressed significantly more NLRP3 protein than circulating monocytes. This study found that TAM NLRP3 expression was controlled by TGF-β *in vivo*, a known secretion product of tumour cells [144]. Similar results were observed in a model of colorectal cancer, where TAMs surrounding the cancer have strong NLRP3 and IL-1β expression, as well as inflammasome activation. These changes in gene expression can be induced by exposure to conditioned medium from colorectal cancer cell line, suggesting that crosstalk between cancer and TAMs drives an inflammasome response in these macrophages [145]. Signal 2 of inflammasome assembly could be provided by ATP, which is commonly elevated at tumour sites [146]. P2X_7_, an important receptor for ATP signalling, is overexpressed in many tumours and has been established as a mediator of inflammatory cytokine release into circulation e.g. IL-1β and TNF-α [147], as well as proteases e.g. matrix metalloproteinase-9 (MMP-9) [148] and cathepsins [149] that could contribute to tumour progression.

In addition to TAMs, cancer-associated fibroblasts (CAFs) account for large proportion of cells within the microenvironment of solid tumours e.g. breast cancer and are associated with poor prognosis [150]. CAFs are also inflammasome forming cells. Ershaid et al. (2019) found that in both mouse and human CAFs, the expression of NLRP3, caspase-1 and IL-1β are significantly upregulated in breast tumour samples [151], strongly suggesting that the tumour environment can prime the inflammasome. Indeed, murine mammary fibroblasts treated with DAMPs (ATP and MSU) responded with increased caspase-1 cleavage and IL-1β secretion [151]. Targeting IL-1β with canakinumab as part of the CANTOS study revealed decreased incidence of lung cancer as well as lower cancer mortality compared to the placebo group [152].

Although combating tumours with radiation can be an effective treatment, it can also result in toxic side effects. One of the most common of these is clinical pneumonitis [153], which leads to lung damage in patients. It is believed that inflammation plays an important role in the pathogenesis of clinical pneumonitis. Indeed, mice irradiated at the thorax experience an increase in macrophages expressing TNF-α, IL-1α and IL-1β in the lung and bronchoalveolar lavage [154]. Murine BMDMs subjected to radiation undergo NLRP3 dependent pyroptosis and secrete elevated levels of IL-1β and IL-18 [155]. *In vivo,* low dose radiation is sufficient to induce NLRP3 expression and inflammasome activation in the mouse lung. Moreover, inhibition or deletion of NLRP3 prevented radiation reduced inflammation score [156]. Therefore, radiation treatment can induce NLRP3 activation in a sterile environment, resulting in death of non-cancerous cells and pro-inflammatory cytokine release. Moreover, in cancer patients diagnosed with painful neuropathy as a side effect of the drug Bortezomib, NLRP3 has been found to be overexpressed as a consequence of acetylation of histone H3 and H4 in the NLRP3 promoter region [157], thus driving the disease. On the other hand, some chemotherapy treatment induced NLRP3 activation has been described as beneficial for battling tumours [158]. It has been reported that anti-breast tumour chemotherapy efficacy relies on myeloid PTEN, which directly interacts with and dephosphorylates NLRP3 to facilitate interaction with ASC and inflammasome activation. Clinical data suggests that myeloid PTEN expression correlates with chemotherapy induced anti-tumour immunity in patients with breast cancer [158]. These studies provide key evidence that transcriptional, post-transcriptional and post-translational licencing of NLRP3 has a role in clinical outcomes.

## 3 Conclusion

Priming has long been thought of as a prerequisite for inflammasome activation, with PAMPs being commonly used to prime NLRP3 via gene upregulation and licence NLRP3 via PTMs *in vitro*. It has becoming increasingly evident that the NLRP3 inflammasome is a key driver in many non-communicable diseases. The process of aging and the pathogenesis of inflammatory conditions associated with aging result in the increase of various pro-inflammatory cytokines, metabolites, aggregates and chemically reactive species. All these have been evidenced to prime the NLRP3 inflammasome via different mechanisms and result in an enhanced inflammasome response, further driving inflammation and disease progression. The distinction between the inflammasome priming and activation steps during the pathogenesis inflammatory conditions is difficult to make. In young, healthy individuals, it remains elusive whether any unknown innate factors prime the inflammasome *in vivo*. Therefore, there is still no universal definition of what a primed and ‘ready to assemble’ inflammasome looks like. Although a number of post-transcriptional and post-translational modifications have been proposed, the landscape of constitutively acting RNAs and enzymes on NLRP3, as well as the sequence of modifications that occur in response to different stimuli and throughout aging remains uncharacterised. Moreover, we do not know which modifications are required *in vivo*, and how these differ between cell types and species. As the majority of studies are carrying out in murine cells, the mechanisms of priming in humans remain largely unstudied. More work must be done before considering targeting NLRP3 modifying enzymes or priming pathways to treat inflammatory conditions.

## Funding

This work is supported by: a Medical Research Council PhD DTP studentship to A.G. (MR/N013751/1); a Presidential Fellowship to J.P.G. (University of Manchester); a 10.13039/501100007155Medical Research Council grant to D.B. (MR/N029992/1) and a Wellcome Trust and Royal Society Henry Dale Fellowship to G.L-C. (104192/Z/14/Z).

## Declaration of Competing Interest

The authors declare no conflict of interest.

## References

[1] Takeuchi O., Akira S. (2010). Pattern recognition receptors and inflammation. Cell.

[2] Kelley N., Jeltema D., Duan Y., He Y. (2019). The NLRP3 inflammasome: an overview of mechanisms of activation and regulation. Int. J. Mol. Sci..

[3] Bauernfeind F.G., Horvath G., Stutz A., Alnemri E.S., MacDonald K., Speert D., Fernandes-Alnemri T., Wu J., Monks B.G., Fitzgerald K.A., Hornung V., Latz E. (2009). Cutting edge: NF-kappaB activating pattern recognition and cytokine receptors license NLRP3 inflammasome activation by regulating NLRP3 expression. J. Immunol..

[4] Shim D.W., Lee K.H. (2018). Posttranslational regulation of the NLR family pyrin domain-containing 3 inflammasome. Front. Immunol..

[5] Han S., Lear T.B., Jerome J.A., Rajbhandari S., Snavely C.A., Gulick D.L., Gibson K.F., Zou C., Chen B.B., Mallampalli R.K. (2015). Lipopolysaccharide primes the NALP3 inflammasome by inhibiting its ubiquitination and degradation mediated by the SCFFBXL2 E3 ligase. J. Biol. Chem..

[6] Broz P., Dixit V.M. (2016). Inflammasomes: mechanism of assembly, regulation and signalling. Nat. Rev. Immunol..

[7] Shi J., Zhao Y., Wang K., Shi X., Wang Y., Huang H., Zhuang Y., Cai T., Wang F., Shao F. (2015). Cleavage of GSDMD by inflammatory caspases determines pyroptotic cell death. Nature.

[8] Gaidt M., Ebert T., Chauhan T., Schmidt D. (2016). Human monocytes engage an alternative inflammasome pathway. Immunity.

[9] Dinarello C.A. (2011). Interleukin-1 in the pathogenesis and treatment of inflammatory diseases. Blood.

[10] Latz E., Duewell P. (2018). NLRP3 inflammasome activation in inflammaging. Semin. Immunol..

[11] Youm Y.H., Grant R.W., McCabe L.R., Albarado D.C., Nguyen K.Y., Ravussin A., Pistell P., Newman S., Carter R., Laque A., Münzberg H., Rosen C.J., Ingram D.K., Salbaum J.M., Dixit V.D. (2013). Canonical Nlrp3 inflammasome links systemic low-grade inflammation to functional decline in aging. Cell Metab..

[12] Anand P.K., Malireddi R.K., Kanneganti T.D. (2011). Role of the nlrp3 inflammasome in microbial infection. Front. Microbiol..

[13] Juliana C., Fernandes-Alnemri T., Kang S., Farias A., Qin F., Alnemri E.S. (2012). Non-transcriptional priming and deubiquitination regulate NLRP3 inflammasome activation. J. Biol. Chem..

[14] Eigenbrod T., Park J.H., Harder J., Iwakura Y., Núñez G. (2008). Cutting edge: critical role for mesothelial cells in necrosis-induced inflammation through the recognition of IL-1 alpha released from dying cells. J. Immunol..

[15] Franchi L., Eigenbrod T., Núñez G. (2009). Cutting edge: TNF-alpha mediates sensitization to ATP and silica via the NLRP3 inflammasome in the absence of microbial stimulation. J. Immunol..

[16] Hiscott J., Marois J., Garoufalis J., D’Addario M., Roulston A., Kwan I., Pepin N., Lacoste J., Nguyen H., Bensi G. (1993). Characterization of a functional NF-kappa B site in the human interleukin 1 beta promoter: evidence for a positive autoregulatory loop. Mol. Cell. Biol..

[17] Anderson J.P., Mueller J.L., Misaghi A., Anderson S., Sivagnanam M., Kolodner R.D., Hoffman H.M. (2008). Initial description of the human NLRP3 promoter. Genes Immun..

[18] Xiao H., Lu M., Lin T.Y., Chen Z., Chen G., Wang W.C., Marin T., Shentu T.P., Wen L., Gongol B., Sun W., Liang X., Chen J., Huang H.D., Pedra J.H., Johnson D.A., Shyy J.Y. (2013). Sterol regulatory element binding protein 2 activation of NLRP3 inflammasome in endothelium mediates hemodynamic-induced atherosclerosis susceptibility. Circulation.

[19] Hoss F., Mueller J.L., Rojas Ringeling F., Rodriguez-Alcazar J.F., Brinkschulte R., Seifert G., Stahl R., Broderick L., Putnam C.D., Kolodner R.D., Canzar S., Geyer M., Hoffman H.M., Latz E. (2019). Alternative splicing regulates stochastic NLRP3 activity. Nat. Commun..

[20] Schmacke N.A., Gaidt M.M., Szymanska I., O’Duill F., Stafford C.A., Chauhan D., Fröhlich A.L., Nagl D., Pinci F., Schmid-Burgk J.L., Hornung V. (2019). Priming enables a NEK7-independent route of NLRP3 activation. bioRxiv.

[21] Bayarsaihan D. (2011). Epigenetic mechanisms in inflammation. J. Dent. Res..

[22] Wei M., Wang L., Wu T., Xi J., Han Y., Yang X., Zhang D., Fang Q., Tang B. (2016). NLRP3 activation was regulated by DNA methylation modification during Mycobacterium tuberculosis infection. Biomed Res. Int..

[23] Coll R.C., O’Neill L.A. (2010). New insights into the regulation of signalling by toll-like receptors and nod-like receptors. J. Innate Immun..

[24] Bauernfeind F., Rieger A., Schildberg F.A., Knolle P.A., Schmid-Burgk J.L., Hornung V. (2012). NLRP3 inflammasome activity is negatively controlled by miR-223. J. Immunol..

[25] Tezcan G., Martynova E.V., Gilazieva Z.E., McIntyre A., Rizvanov A.A., Khaiboullina S.F. (2019). MicroRNA post-transcriptional regulation of the NLRP3 inflammasome in Immunopathologies. Front. Pharmacol..

[26] Haneklaus M., Gerlic M., Kurowska-Stolarska M., Rainey A.A., Pich D., McInnes I.B., Hammerschmidt W., O’Neill L.A., Masters S.L. (2012). Cutting edge: miR-223 and EBV miR-BART15 regulate the NLRP3 inflammasome and IL-1β production. J. Immunol..

[27] Haneklaus M., O’Neil J.D., Clark A.R., Masters S.L., O’Neill L.A.J. (2017). The RNA-binding protein Tristetraprolin (TTP) is a critical negative regulator of the NLRP3 inflammasome. J. Biol. Chem..

[28] Millar A.H., Heazlewood J.L., Giglione C., Holdsworth M.J., Bachmair A., Schulze W.X., Scope The (2019). Functions, and dynamics of posttranslational protein modifications. Annu. Rev. Plant Biol..

[29] Lopez-Castejon G. (2020). Control of the inflammasome by the ubiquitin system. FEBS J..

[30] Song N., Li T. (2018). Regulation of NLRP3 inflammasome by phosphorylation. Front. Immunol..

[31] Song N., Liu Z.S., Xue W., Bai Z.F., Wang Q.Y., Dai J., Liu X., Huang Y.J., Cai H., Zhan X.Y., Han Q.Y., Wang H., Chen Y., Li H.Y., Li A.L., Zhang X.M., Zhou T., Li T. (2017). NLRP3 phosphorylation is an essential priming event for inflammasome activation. Mol. Cell.

[32] Tang J., Tu S., Lin G., Guo H., Yan C., Liu Q., Huang L., Tang N., Xiao Y., Pope R.M., Rajaram M.V.S., Amer A.O., Ahmer B.M., Gunn J.S., Wozniak D.J., Tao L., Coppola V., Zhang L., Langdon W.Y., Torrelles J.B., Lipkowitz S., Zhang J. (2020). Sequential ubiquitination of NLRP3 by RNF125 and Cbl-b limits inflammasome activation and endotoxemia. J. Exp. Med..

[33] Duong B.H., Onizawa M., Oses-Prieto J.A., Advincula R., Burlingame A., Malynn B.A., Ma A. (2015). A20 restricts ubiquitination of pro-interleukin-1β protein complexes and suppresses NLRP3 inflammasome activity. Immunity.

[34] Humphries F., Bergin R., Jackson R., Delagic N., Wang B., Yang S., Dubois A.V., Ingram R.J., Moynagh P.N. (2018). The E3 ubiquitin ligase Pellino2 mediates priming of the NLRP3 inflammasome. Nat. Commun..

[35] Zhao K., Zhang Y., Xu X., Liu L., Huang L., Luo R., Li J., Zhang N., Lu B. (2019). Acetylation is required for NLRP3 self-aggregation and full activation of the inflammasome. BioRXiv.

[36] He M., Chiang H.H., Luo H., Zheng Z., Qiao Q., Wang L., Tan M., Ohkubo R., Mu W.C., Zhao S., Wu H., Chen D. (2020). An acetylation switch of the NLRP3 inflammasome regulates aging-associated chronic inflammation and insulin resistance. Cell Metab..

[37] Chung H.Y., Kim D.H., Lee E.K., Chung K.W., Chung S., Lee B., Seo A.Y., Chung J.H., Jung Y.S., Im E., Lee J., Kim N.D., Choi Y.J., Im D.S., Yu B.P. (2019). Redefining chronic inflammation in aging and age-related diseases: proposal of the senoinflammation concept. Aging Dis..

[38] Jura M., Kozak L.P. (2016). Obesity and related consequences to ageing. Age (Dordr).

[39] Takahashi R., Totsuka S., Ishigami A., Kobayashi Y., Nagata K. (2016). Attenuated phagocytosis of secondary necrotic neutrophils by macrophages in aged and SMP30 knockout mice. Geriatr. Gerontol. Int..

[40] Mejías-Peña Y., Rodriguez-Miguelez P., Fernandez-Gonzalo R., Martínez-Flórez S., Almar M., de Paz J.A., Cuevas M.J., González-Gallego J. (2016). Effects of aerobic training on markers of autophagy in the elderly. Age (Dordr).

[41] Pyo J.O., Yoo S.M., Ahn H.H., Nah J., Hong S.H., Kam T.I., Jung S., Jung Y.K. (2013). Overexpression of Atg5 in mice activates autophagy and extends lifespan. Nat. Commun..

[42] Herbig U., Ferreira M., Condel L., Carey D., Sedivy J.M. (2006). Cellular senescence in aging primates. Science.

[43] Sohal R.S., Sohal B.H. (1991). Hydrogen peroxide release by mitochondria increases during aging. Mech. Ageing Dev..

[44] Thevaranjan N., Puchta A., Schulz C., Naidoo A., Szamosi J.C., Verschoor C.P., Loukov D., Schenck L.P., Jury J., Foley K.P., Schertzer J.D., Larché M.J., Davidson D.J., Verdú E.F., Surette M.G., Bowdish D.M.E. (2017). Age-associated microbial dysbiosis promotes intestinal permeability, systemic inflammation, and macrophage dysfunction. Cell Host Microbe.

[45] Gombar S., Jung H.J., Dong F., Calder B., Atzmon G., Barzilai N., Tian X.L., Pothof J., Hoeijmakers J.H., Campisi J., Vijg J., Suh Y. (2012). Comprehensive microRNA profiling in B-cells of human centenarians by massively parallel sequencing. BMC Genomics.

[46] van Deursen J.M. (2014). The role of senescent cells in ageing. Nature.

[47] Watanabe S., Kawamoto S., Ohtani N., Hara E. (2017). Impact of senescence-associated secretory phenotype and its potential as a therapeutic target for senescence-associated diseases. Cancer Sci..

[48] Rodier F., Coppé J.P., Patil C.K., Hoeijmakers W.A., Muñoz D.P., Raza S.R., Freund A., Campeau E., Davalos A.R., Campisi J. (2009). Persistent DNA damage signalling triggers senescence-associated inflammatory cytokine secretion. Nat. Cell Biol..

[49] Yang H., Wang H., Ren J., Chen Q., Chen Z.J. (2017). cGAS is essential for cellular senescence. Proc. Natl. Acad. Sci. U. S. A..

[50] Han X., Chen H., Gong H., Tang X., Huang N., Xu W., Tai H., Zhang G., Zhao T., Gong C., Wang S., Yang Y., Xiao H. (2020). Autolysosomal degradation of cytosolic chromatin fragments antagonizes oxidative stress-induced senescence. J. Biol. Chem..

[51] Chien Y., Scuoppo C., Wang X., Fang X., Balgley B., Bolden J.E., Premsrirut P., Luo W., Chicas A., Lee C.S., Kogan S.C., Lowe S.W. (2011). Control of the senescence-associated secretory phenotype by NF-κB promotes senescence and enhances chemosensitivity. Genes Dev..

[52] Freund A., Orjalo A.V., Desprez P.Y., Campisi J. (2010). Inflammatory networks during cellular senescence: causes and consequences. Trends Mol. Med..

[53] Temmoku J., Fujita Y., Matsuoka N., Urano T., Furuya M.Y., Asano T., Sato S., Matsumoto H., Watanabe H., Kozuru H., Yatsuhashi H., Kawakami A., Migita K. (2020). Uric acid-mediated inflammasome activation in IL-6 primed innate immune cells is regulated by baricitinib. Mod. Rheumatol..

[54] Hall B.M., Balan V., Gleiberman A.S., Strom E., Krasnov P., Virtuoso L.P., Rydkina E., Vujcic S., Balan K., Gitlin I., Leonova K., Polinsky A., Chernova O.B., Gudkov A.V. (2016). Aging of mice is associated with p16(Ink4a)- and β-galactosidase-positive macrophage accumulation that can be induced in young mice by senescent cells. Aging (Albany NY).

[55] Stout-Delgado H.W., Cho S.J., Chu S.G., Mitzel D.N., Villalba J., El-Chemaly S., Ryter S.W., Choi A.M., Rosas I.O. (2016). Age-dependent susceptibility to pulmonary fibrosis is associated with NLRP3 inflammasome activation. Am. J. Respir. Cell Mol. Biol..

[56] Acosta J.C., Banito A., Wuestefeld T., Georgilis A., Janich P., Morton J.P., Athineos D., Kang T.W., Lasitschka F., Andrulis M., Pascual G., Morris K.J., Khan S., Jin H., Dharmalingam G., Snijders A.P., Carroll T., Capper D., Pritchard C., Inman G.J., Longerich T., Sansom O.J., Benitah S.A., Zender L., Gil J. (2013). A complex secretory program orchestrated by the inflammasome controls paracrine senescence. Nat. Cell Biol..

[57] Segula D. (2014). Complications of obesity in adults: a short review of the literature. Malawi Med. J..

[58] Rochlani Y., Pothineni N.V., Kovelamudi S., Mehta J.L. (2017). Metabolic syndrome: pathophysiology, management, and modulation by natural compounds. Ther. Adv. Cardiovasc. Dis..

[59] Vishram J.K., Borglykke A., Andreasen A.H., Jeppesen J., Ibsen H., Jørgensen T., Palmieri L., Giampaoli S., Donfrancesco C., Kee F., Mancia G., Cesana G., Kuulasmaa K., Salomaa V., Sans S., Ferrieres J., Dallongeville J., Söderberg S., Arveiler D., Wagner A., Tunstall-Pedoe H., Drygas W., Olsen M.H., Project M. (2014). Impact of age and gender on the prevalence and prognostic importance of the metabolic syndrome and its components in Europeans. The MORGAM Prospective Cohort Project. PLoS One.

[60] Kotani K., Tokunaga K., Fujioka S., Kobatake T., Keno Y., Yoshida S., Shimomura I., Tarui S., Matsuzawa Y. (1994). Sexual dimorphism of age-related changes in whole-body fat distribution in the obese. Int. J. Obes. Relat. Metab. Disord..

[61] Singh G.M., Danaei G., Pelizzari P.M., Lin J.K., Cowan M.J., Stevens G.A., Farzadfar F., Khang Y.H., Lu Y., Riley L.M., Lim S.S., Ezzati M. (2012). The age associations of blood pressure, cholesterol, and glucose: analysis of health examination surveys from international populations. Circulation.

[62] Bournat J.C., Brown C.W. (2010). Mitochondrial dysfunction in obesity. Curr. Opin. Endocrinol. Diabetes Obes..

[63] Cortopassi G.A., Arnheim N. (1990). Detection of a specific mitochondrial DNA deletion in tissues of older humans. Nucleic Acids Res..

[64] Mori M.A., Raghavan P., Thomou T., Boucher J., Robida-Stubbs S., Macotela Y., Russell S.J., Kirkland J.L., Blackwell T.K., Kahn C.R. (2012). Role of microRNA processing in adipose tissue in stress defense and longevity. Cell Metab..

[65] Bauernfeind F., Bartok E., Rieger A., Franchi L., Núñez G., Hornung V. (2011). Cutting edge: reactive oxygen species inhibitors block priming, but not activation, of the NLRP3 inflammasome. J. Immunol..

[66] Rathore R., Zheng Y.M., Niu C.F., Liu Q.H., Korde A., Ho Y.S., Wang Y.X. (2008). Hypoxia activates NADPH oxidase to increase [ROS]i and [Ca2+]i through the mitochondrial ROS-PKCepsilon signaling axis in pulmonary artery smooth muscle cells. Free Radic. Biol. Med..

[67] Panchanathan R., Liu H., Choubey D. (2016). Hypoxia primes human normal prostate epithelial cells and cancer cell lines for the NLRP3 and AIM2 inflammasome activation. Oncotarget.

[68] Folco E.J., Sukhova G.K., Quillard T., Libby P. (2014). Moderate hypoxia potentiates interleukin-1β production in activated human macrophages. Circ. Res..

[69] Al-Goblan A.S., Al-Alfi M.A., Khan M.Z. (2014). Mechanism linking diabetes mellitus and obesity. Diabetes Metab. Syndr. Obes..

[70] Morley J.E. (2008). Diabetes and aging: epidemiologic overview. Clin. Geriatr. Med..

[71] Bauernfeind F., Niepmann S., Knolle P.A., Hornung V. (2016). Aging-associated TNF production primes inflammasome activation and NLRP3-Related metabolic disturbances. J. Immunol..

[72] Weisberg S.P., McCann D., Desai M., Rosenbaum M., Leibel R.L., Ferrante A.W. (2003). Obesity is associated with macrophage accumulation in adipose tissue. J. Clin. Invest..

[73] Ringseis R., Eder K., Mooren F.C., Krüger K. (2015). Metabolic signals and innate immune activation in obesity and exercise. Exerc. Immunol. Rev..

[74] Shi H., Kokoeva M.V., Inouye K., Tzameli I., Yin H., Flier J.S. (2006). TLR4 links innate immunity and fatty acid-induced insulin resistance. J. Clin. Invest..

[75] De Boer A.A., Monk J.M., Liddle D.M., Hutchinson A.L., Power K.A., Ma D.W., Robinson L.E. (2016). Fish-oil-derived n-3 polyunsaturated fatty acids reduce NLRP3 inflammasome activity and obesity-related inflammatory cross-talk between adipocytes and CD11b(+) macrophages. J. Nutr. Biochem..

[76] Finucane O.M., Lyons C.L., Murphy A.M., Reynolds C.M., Klinger R., Healy N.P., Cooke A.A., Coll R.C., McAllan L., Nilaweera K.N., O’Reilly M.E., Tierney A.C., Morine M.J., Alcala-Diaz J.F., Lopez-Miranda J., O’Connor D.P., O’Neill L.A., McGillicuddy F.C., Roche H.M. (2015). Monounsaturated fatty acid-enriched high-fat diets impede adipose NLRP3 inflammasome-mediated IL-1β secretion and insulin resistance despite obesity. Diabetes.

[77] Snodgrass R.G., Huang S., Choi I.W., Rutledge J.C., Hwang D.H. (2013). Inflammasome-mediated secretion of IL-1β in human monocytes through TLR2 activation; modulation by dietary fatty acids. J. Immunol..

[78] Adiels M., Taskinen M.R., Björnson E., Andersson L., Matikainen N., Söderlund S., Kahri J., Hakkarainen A., Lundbom N., Sihlbom C., Thorsell A., Zhou H., Pietiläinen K.H., Packard C., Borén J. (2019). Role of apolipoprotein C-III overproduction in diabetic dyslipidaemia. Diabetes Obes. Metab..

[79] Raposo H.F., Paiva A.A., Kato L.S., de Oliveira H.C. (2015). Apolipoprotein CIII overexpression exacerbates diet-induced obesity due to adipose tissue higher exogenous lipid uptake and retention and lower lipolysis rates. Nutr Metab (Lond).

[80] Zewinger S., Reiser J., Jankowski V., Alansary D., Hahm E., Triem S., Klug M., Schunk S.J., Schmit D., Kramann R., Körbel C., Ampofo E., Laschke M.W., Selejan S.R., Paschen A., Herter T., Schuster S., Silbernagel G., Sester M., Sester U., Aßmann G., Bals R., Kostner G., Jahnen-Dechent W., Menger M.D., Rohrer L., März W., Böhm M., Jankowski J., Kopf M., Latz E., Niemeyer B.A., Fliser D., Laufs U., Speer T. (2020). Apolipoprotein C3 induces inflammation and organ damage by alternative inflammasome activation. Nat. Immunol..

[81] Singh V.P., Bali A., Singh N., Jaggi A.S. (2014). Advanced glycation end products and diabetic complications. Korean J. Physiol. Pharmacol..

[82] Amin M.N., Mosa A.A., El-Shishtawy M.M. (2011). Clinical study of advanced glycation end products in egyptian diabetic obese and non-obese patients. Int. J. Biomed. Sci..

[83] Chen J., Sun Z., Jin M., Tu Y., Wang S., Yang X., Chen Q., Zhang X., Han Y., Pi R. (2017). Inhibition of AGEs/RAGE/Rho/ROCK pathway suppresses non-specific neuroinflammation by regulating BV2 microglial M1/M2 polarization through the NF-κB pathway. J. Neuroimmunol..

[84] Kong X., Lu A.L., Yao X.M., Hua Q., Li X.Y., Qin L., Zhang H.M., Meng G.X., Su Q. (2017). Activation of NLRP3 inflammasome by advanced glycation end products promotes pancreatic islet damage. Oxid. Med. Cell. Longev..

[85] Eklund K.K., Niemi K., Kovanen P.T. (2012). Immune functions of serum amyloid A. Crit. Rev. Immunol..

[86] Rosenthal C.J., Franklin E.C. (1975). Variation with age and disease of an amyloid A protein-related serum component. J. Clin. Invest..

[87] Marzi C., Huth C., Herder C., Baumert J., Thorand B., Rathmann W., Meisinger C., Wichmann H.E., Roden M., Peters A., Grallert H., Koenig W., Illig T. (2013). Acute-phase serum amyloid A protein and its implication in the development of type 2 diabetes in the KORA S4/F4 study. Diabetes Care.

[88] Savage C.D., Lopez-Castejon G., Denes A., Brough D. (2012). NLRP3-inflammasome activating DAMPs stimulate an inflammatory response in Glia in the absence of priming which contributes to brain inflammation after injury. Front. Immunol..

[89] Niemi K., Teirilä L., Lappalainen J., Rajamäki K., Baumann M.H., Öörni K., Wolff H., Kovanen P.T., Matikainen S., Eklund K.K. (2011). Serum amyloid A activates the NLRP3 inflammasome via P2X7 receptor and a cathepsin B-sensitive pathway. J. Immunol..

[90] Shridas P., De Beer M.C., Webb N.R. (2018). High-density lipoprotein inhibits serum amyloid A-mediated reactive oxygen species generation and NLRP3 inflammasome activation. J. Biol. Chem..

[91] Rauscher F.M., Goldschmidt-Clermont P.J., Davis B.H., Wang T., Gregg D., Ramaswami P., Pippen A.M., Annex B.H., Dong C., Taylor D.A. (2003). Aging, progenitor cell exhaustion, and atherosclerosis. Circulation.

[92] Libby P. (2002). Inflammation in atherosclerosis. Nature.

[93] Zhang L., Connelly J.J., Peppel K., Brian L., Shah S.H., Nelson S., Crosslin D.R., Wang T., Allen A., Kraus W.E., Gregory S.G., Hauser E.R., Freedman N.J. (2010). Aging-related atherosclerosis is exacerbated by arterial expression of tumor necrosis factor receptor-1: evidence from mouse models and human association studies. Hum. Mol. Genet..

[94] Wright S.D., Burton C., Hernandez M., Hassing H., Montenegro J., Mundt S., Patel S., Card D.J., Hermanowski-Vosatka A., Bergstrom J.D., Sparrow C.P., Detmers P.A., Chao Y.S. (2000). Infectious agents are not necessary for murine atherogenesis. J. Exp. Med..

[95] Rhoads J.P., Major A.S. (2018). How oxidized low-density lipoprotein activates inflammatory responses. Crit. Rev. Immunol..

[96] Park Y.M. (2014). CD36, a scavenger receptor implicated in atherosclerosis. Exp. Mol. Med..

[97] Sheedy F.J., Grebe A., Rayner K.J., Kalantari P., Ramkhelawon B., Carpenter S.B., Becker C.E., Ediriweera H.N., Mullick A.E., Golenbock D.T., Stuart L.M., Latz E., Fitzgerald K.A., Moore K.J. (2013). CD36 coordinates NLRP3 inflammasome activation by facilitating intracellular nucleation of soluble ligands into particulate ligands in sterile inflammation. Nat. Immunol..

[98] Menu P., Pellegrin M., Aubert J.F., Bouzourene K., Tardivel A., Mazzolai L., Tschopp J. (2011). Atherosclerosis in ApoE-deficient mice progresses independently of the NLRP3 inflammasome. Cell Death Dis..

[99] Christ A., Günther P., Lauterbach M.A.R., Duewell P., Biswas D., Pelka K., Scholz C.J., Oosting M., Haendler K., Baßler K., Klee K., Schulte-Schrepping J., Ulas T., Moorlag S.J.C.F., Kumar V., Park M.H., Joosten L.A.B., Groh L.A., Riksen N.P., Espevik T., Schlitzer A., Li Y., Fitzgerald M.L., Netea M.G., Schultze J.L., Latz E. (2018). Western diet triggers NLRP3-Dependent innate immune reprogramming. Cell.

[100] Karasawa T., Takahashi M. (2017). Role of NLRP3 inflammasomes in atherosclerosis. J. Atheroscler. Thromb..

[101] Ridker P.M., Everett B.M., Thuren T., MacFadyen J.G., Chang W.H., Ballantyne C., Fonseca F., Nicolau J., Koenig W., Anker S.D., Kastelein J.J.P., Cornel J.H., Pais P., Pella D., Genest J., Cifkova R., Lorenzatti A., Forster T., Kobalava Z., Vida-Simiti L., Flather M., Shimokawa H., Ogawa H., Dellborg M., Rossi P.R.F., Troquay R.P.T., Libby P., Glynn R.J., Group C.T. (2017). Antiinflammatory therapy with canakinumab for atherosclerotic disease. N. Engl. J. Med..

[102] Duewell P., Kono H., Rayner K.J., Sirois C.M., Vladimer G., Bauernfeind F.G., Abela G.S., Franchi L., Nuñez G., Schnurr M., Espevik T., Lien E., Fitzgerald K.A., Rock K.L., Moore K.J., Wright S.D., Hornung V., Latz E. (2010). NLRP3 inflammasomes are required for atherogenesis and activated by cholesterol crystals. Nature.

[103] Rajamäki K., Lappalainen J., Oörni K., Välimäki E., Matikainen S., Kovanen P.T., Eklund K.K. (2010). Cholesterol crystals activate the NLRP3 inflammasome in human macrophages: a novel link between cholesterol metabolism and inflammation. PLoS One.

[104] Wen C., Yang X., Yan Z., Zhao M., Yue X., Cheng X., Zheng Z., Guan K., Dou J., Xu T., Zhang Y., Song T., Wei C., Zhong H. (2013). Nalp3 inflammasome is activated and required for vascular smooth muscle cell calcification. Int. J. Cardiol..

[105] Guo R.F., Ward P.A. (2005). Role of C5a in inflammatory responses. Annu. Rev. Immunol..

[106] Gaya da Costa M., Poppelaars F., van Kooten C., Mollnes T.E., Tedesco F., Würzner R., Trouw L.A., Truedsson L., Daha M.R., Roos A., Seelen M.A. (2018). Age and sex-associated changes of complement activity and complement levels in a healthy caucasian population. Front. Immunol..

[107] Wezel A., de Vries M.R., Lagraauw H.M., Foks A.C., Kuiper J., Quax P.H., Bot I. (2014). Complement factor C5a induces atherosclerotic plaque disruptions. J. Cell. Mol. Med..

[108] Samstad E.O., Niyonzima N., Nymo S., Aune M.H., Ryan L., Bakke S.S., Lappegård K.T., Brekke O.L., Lambris J.D., Damås J.K., Latz E., Mollnes T.E., Espevik T. (2014). Cholesterol crystals induce complement-dependent inflammasome activation and cytokine release. J. Immunol..

[109] Haggadone M.D., Grailer J.J., Fattahi F., Zetoune F.S., Ward P.A. (2016). Bidirectional crosstalk between C5a receptors and the NLRP3 inflammasome in macrophages and monocytes. Mediators Inflamm..

[110] Hou Y., Dan X., Babbar M., Wei Y., Hasselbalch S.G., Croteau D.L., Bohr V.A. (2019). Ageing as a risk factor for neurodegenerative disease. Nat. Rev. Neurol..

[111] Louveau A., Harris T.H., Kipnis J. (2015). Revisiting the mechanisms of CNS immune privilege. Trends Immunol..

[112] Yang A.C., Stevens M.Y., Chen M.B., Lee D.P., Stähli D., Gate D., Contrepois K., Chen W., Iram T., Zhang L., Vest R.T., Chaney A., Lehallier B., Olsson N., du Bois H., Hsieh R., Cropper H.C., Berdnik D., Li L., Wang E.Y., Traber G.M., Bertozzi C.R., Luo J., Snyder M.P., Elias J.E., Quake S.R., James M.L., Wyss-Coray T. (2020). Physiological blood-brain transport is impaired with age by a shift in transcytosis. Nature.

[113] Cribbs D.H., Berchtold N.C., Perreau V., Coleman P.D., Rogers J., Tenner A.J., Cotman C.W. (2012). Extensive innate immune gene activation accompanies brain aging, increasing vulnerability to cognitive decline and neurodegeneration: a microarray study. J. Neuroinflammation.

[114] Komatsu M., Waguri S., Chiba T., Murata S., Iwata J., Tanida I., Ueno T., Koike M., Uchiyama Y., Kominami E., Tanaka K. (2006). Loss of autophagy in the central nervous system causes neurodegeneration in mice. Nature.

[115] Mputhia Z., Hone E., Tripathi T., Sargeant T., Martins R., Bharadwaj P. (2019). Autophagy modulation as a treatment of amyloid diseases. Molecules.

[116] Qing G., Yan P., Qu Z., Liu H., Xiao G. (2007). Hsp90 regulates processing of NF-kappa B2 p100 involving protection of NF-kappa B-inducing kinase (NIK) from autophagy-mediated degradation. Cell Res..

[117] Xia X., Jiang Q., McDermott J., Han J.J. (2018). Aging and Alzheimer’s disease: comparison and associations from molecular to system level. Aging Cell.

[118] Voet S., Mc Guire C., Hagemeyer N., Martens A., Schroeder A., Wieghofer P., Daems C., Staszewski O., Vande Walle L., Jordao M.J.C., Sze M., Vikkula H.K., Demeestere D., Van Imschoot G., Scott C.L., Hoste E., Gonçalves A., Guilliams M., Lippens S., Libert C., Vandenbroucke R.E., Kim K.W., Jung S., Callaerts-Vegh Z., Callaerts P., de Wit J., Lamkanfi M., Prinz M. (2018). G. Van Loo, A20 critically controls microglia activation and inhibits inflammasome-dependent neuroinflammation. Nat. Commun..

[119] Nizami S., Hall-Roberts H., Warrier S., Cowley S.A., Di Daniel E. (2019). Microglial inflammation and phagocytosis in Alzheimer’s disease: potential therapeutic targets. Br. J. Pharmacol..

[120] Li Y., Liu L., Barger S.W., Griffin W.S. (2003). Interleukin-1 mediates pathological effects of microglia on tau phosphorylation and on synaptophysin synthesis in cortical neurons through a p38-MAPK pathway. J. Neurosci..

[121] Nakanishi A., Kaneko N., Takeda H., Sawasaki T., Morikawa S., Zhou W., Kurata M., Yamamoto T., Akbar S.M.F., Zako T., Masumoto J. (2018). Amyloid β directly interacts with NLRP3 to initiate inflammasome activation: identification of an intrinsic NLRP3 ligand in a cell-free system. Inflamm. Regen..

[122] Hughes C., Choi M.L., Yi J.H., Kim S.C., Drews A., George-Hyslop P.S., Bryant C., Gandhi S., Cho K., Klenerman D. (2020). Beta amyloid aggregates induce sensitised TLR4 signalling causing long-term potentiation deficit and rat neuronal cell death. Commun Biol.

[123] Heneka M.T., Kummer M.P., Stutz A., Delekate A., Schwartz S., Vieira-Saecker A., Griep A., Axt D., Remus A., Tzeng T.C., Gelpi E., Halle A., Korte M., Latz E., Golenbock D.T. (2013). NLRP3 is activated in Alzheimer’s disease and contributes to pathology in APP/PS1 mice. Nature.

[124] Breydo L., Wu J.W., Uversky V.N. (2012). A-synuclein misfolding and Parkinson’s disease. Biochim. Biophys. Acta.

[125] Béraud D., Twomey M., Bloom B., Mittereder A., Ton V., Neitzke K., Chasovskikh S., Mhyre T.R., Maguire-Zeiss K.A. (2011). Α-synuclein alters toll-like receptor expression. Front. Neurosci..

[126] Codolo G., Plotegher N., Pozzobon T., Brucale M., Tessari I., Bubacco L., de Bernard M. (2013). Triggering of inflammasome by aggregated α-synuclein, an inflammatory response in synucleinopathies. PLoS One.

[127] Su X., Maguire-Zeiss K.A., Giuliano R., Prifti L., Venkatesh K., Federoff H.J. (2008). Synuclein activates microglia in a model of Parkinson’s disease. Neurobiol. Aging.

[128] Fan Z., Pan Y.T., Zhang Z.Y., Yang H., Yu S.Y., Zheng Y., Ma J.H., Wang X.M. (2020). Systemic activation of NLRP3 inflammasome and plasma α-synuclein levels are correlated with motor severity and progression in Parkinson’s disease. J. Neuroinflammation.

[129] gov.uk (2020). Multiple Sclerosis: Prevalence, Incidence and Smoking Status - Data Briefing. https://www.gov.uk/government/publications/multiple-sclerosis-prevalence-incidence-and-smoking-status/multiple-sclerosis-prevalence-incidence-and-smoking-status-data.

[130] Musella A., Gentile A., Rizzo F.R., De Vito F., Fresegna D., Bullitta S., Vanni V., Guadalupi L., Stampanoni Bassi M., Buttari F., Centonze D., Mandolesi G. (2018). Interplay between age and neuroinflammation in multiple sclerosis: effects on motor and cognitive functions. Front. Aging Neurosci..

[131] Confavreux C., Vukusic S. (2006). Age at disability milestones in multiple sclerosis. Brain.

[132] Inoue M., Shinohara M.L. (2013). NLRP3 inflammasome and MS/EAE. Autoimmune Dis..

[133] Furlan R., Filippi M., Bergami A., Rocca M.A., Martinelli V., Poliani P.L., Grimaldi L.M., Desina G., Comi G., Martino G. (1999). Peripheral levels of caspase-1 mRNA correlate with disease activity in patients with multiple sclerosis; a preliminary study. J. Neurol. Neurosurg. Psychiatry.

[134] Gris D., Ye Z., Iocca H.A., Wen H., Craven R.R., Gris P., Huang M., Schneider M., Miller S.D., Ting J.P. (2010). NLRP3 plays a critical role in the development of experimental autoimmune encephalomyelitis by mediating Th1 and Th17 responses. J. Immunol..

[135] Inoue M., Williams K.L., Oliver T., Vandenabeele P., Rajan J.V., Miao E.A., Shinohara M.L. (2012). Interferon-β therapy against EAE is effective only when development of the disease depends on the NLRP3 inflammasome. Sci. Signal..

[136] Furlan R., Martino G., Galbiati F., Poliani P.L., Smiroldo S., Bergami A., Desina G., Comi G., Flavell R., Su M.S., Adorini L. (1999). Caspase-1 regulates the inflammatory process leading to autoimmune demyelination. J. Immunol..

[137] Aunan J.R., Cho W.C., Søreide K. (2017). The biology of aging and Cancer: a brief overview of shared and divergent molecular hallmarks. Aging Dis..

[138] Singh N., Baby D., Rajguru J.P., Patil P.B., Thakkannavar S.S., Pujari V.B. (2019). Inflammation and cancer. Ann. Afr. Med..

[139] Coussens L.M., Werb Z. (2002). Inflammation and cancer. Nature.

[140] van Dalen F.J., van Stevendaal M.H.M.E., Fennemann F.L., Verdoes M., Ilina O. (2018). Molecular repolarisation of tumour-associated macrophages. Molecules.

[141] Grivennikov S., Karin E., Terzic J., Mucida D., Yu G.Y., Vallabhapurapu S., Scheller J., Rose-John S., Cheroutre H., Eckmann L., Karin M. (2009). IL-6 and Stat3 are required for survival of intestinal epithelial cells and development of colitis-associated cancer. Cancer Cell.

[142] Alvarez S.E., Harikumar K.B., Hait N.C., Allegood J., Strub G.M., Kim E.Y., Maceyka M., Jiang H., Luo C., Kordula T., Milstien S., Spiegel S. (2010). Sphingosine-1-phosphate is a missing cofactor for the E3 ubiquitin ligase TRAF2. Nature.

[143] Weichand B., Popp R., Dziumbla S., Mora J., Strack E., Elwakeel E., Frank A.C., Scholich K., Pierre S., Syed S.N., Olesch C., Ringleb J., Ören B., Döring C., Savai R., Jung M., von Knethen A., Levkau B., Fleming I., Weigert A., Brüne B. (2017). S1PR1 on tumor-associated macrophages promotes lymphangiogenesis and metastasis via NLRP3/IL-1β. J. Exp. Med..

[144] Daley D., Mani V.R., Mohan N., Akkad N., Pandian G.S.D.B., Savadkar S., Lee K.B., Torres-Hernandez A., Aykut B., Diskin B., Wang W., Farooq M.S., Mahmud A.I., Werba G., Morales E.J., Lall S., Wadowski B.J., Rubin A.G., Berman M.E., Narayanan R., Hundeyin M., Miller G. (2017). NLRP3 signaling drives macrophage-induced adaptive immune suppression in pancreatic carcinoma. J. Exp. Med..

[145] Deng Q., Geng Y., Zhao L., Li R., Zhang Z., Li K., Liang R., Shao X., Huang M., Zuo D., Wu Y., Ma Q. (2019). NLRP3 inflammasomes in macrophages drive colorectal cancer metastasis to the liver. Cancer Lett..

[146] Pellegatti P., Raffaghello L., Bianchi G., Piccardi F., Pistoia V., Di Virgilio F. (2008). Increased level of extracellular ATP at tumor sites: in vivo imaging with plasma membrane luciferase. PLoS One.

[147] De Marchi E., Orioli E., Pegoraro A., Sangaletti S., Portararo P., Curti A., Colombo M.P., Di Virgilio F., Adinolfi E. (2019). The P2X7 receptor modulates immune cells infiltration, ectonucleotidases expression and extracellular ATP levels in the tumor microenvironment. Oncogene.

[148] Gu B.J., Wiley J.S. (2006). Rapid ATP-induced release of matrix metalloproteinase 9 is mediated by the P2X7 receptor. Blood.

[149] Lopez-Castejon G., Theaker J., Pelegrin P., Clifton A.D., Braddock M., Surprenant A. (2010). P2X(7) receptor-mediated release of cathepsins from macrophages is a cytokine-independent mechanism potentially involved in joint diseases. J. Immunol..

[150] Liu L., Yao H.H., Zhu Z.Q., Ning Z.L., Huang Q. (2016). Stromal myofibroblasts are associated with poor prognosis in solid cancers: a meta-analysis of published studies. PLoS One.

[151] Ershaid N., Sharon Y., Doron H., Raz Y., Shani O., Cohen N., Monteran L., Leider-Trejo L., Ben-Shmuel A., Yassin M., Gerlic M., Ben-Baruch A., Pasmanik-Chor M., Apte R., Erez N. (2019). NLRP3 inflammasome in fibroblasts links tissue damage with inflammation in breast cancer progression and metastasis. Nat. Commun..

[152] Ridker P.M., MacFadyen J.G., Thuren T., Everett B.M., Libby P., Glynn R.J., Group C.T. (2017). Effect of interleukin-1β inhibition with canakinumab on incident lung cancer in patients with atherosclerosis: exploratory results from a randomised, double-blind, placebo-controlled trial. Lancet.

[153] Jain V., Berman A.T. (2018). Radiation pneumonitis: old problem, new tricks. Cancers (Basel).

[154] Hong J.H., Jung S.M., Tsao T.C., Wu C.J., Lee C.Y., Chen F.H., Hsu C.H., McBride W.H., Chiang C.S. (2003). Bronchoalveolar lavage and interstitial cells have different roles in radiation-induced lung injury. Int. J. Radiat. Biol..

[155] Liu Y.G., Chen J.K., Zhang Z.T., Ma X.J., Chen Y.C., Du X.M., Liu H., Zong Y., Lu G.C. (2017). NLRP3 inflammasome activation mediates radiation-induced pyroptosis in bone marrow-derived macrophages. Cell Death Dis..

[156] Li X., Gong Y., Li D., Xiang L., Ou Y., Jiang L., Shu P., Liu X., Guo F., Qin D., Mo Z., Qin Q., Wang X., Wang Y. (2020). Low-dose radiation therapy promotes radiation pneumonitis by activating NLRP3 inflammasome. Int. J. Radiat. Oncol. Biol. Phys..

[157] Liu C.C., Huang Z.X., Li X., Shen K.F., Liu M., Ouyang H.D., Zhang S.B., Ruan Y.T., Zhang X.L., Wu S.L., Xin W.J., Ma C. (2018). Upregulation of NLRP3 via STAT3-dependent histone acetylation contributes to painful neuropathy induced by bortezomib. Exp. Neurol..

[158] Huang Y., Wang H., Hao Y., Lin H., Dong M., Ye J., Song L., Wang Y., Li Q., Shan B., Jiang Y., Li H., Shao Z., Kroemer G., Zhang H., Bai L., Jin T., Wang C., Ma Y., Cai Y., Ding C., Liu S., Pan Y., Jiang W., Zhou R. (2020). Myeloid PTEN promotes chemotherapy-induced NLRP3-inflammasome activation and antitumour immunity. Nat. Cell Biol..

